# Evaluation of Atopic Dermatitis and Cutaneous Infectious Disorders Using Sequential Pattern Mining: A Nationwide Population-Based Cohort Study

**DOI:** 10.3390/jcm11123422

**Published:** 2022-06-14

**Authors:** Ju-Hee Han, Jae-Woong Yoon, Hwa-Jung Yook, Chul-Hwan Bang, Jae-Hun Chun, Jun-Young Lee, Young-Min Park, Suk-Jun Lee, Ji-Hyun Lee

**Affiliations:** 1 Department of Dermatology, Seoul St. Mary’s Hospital, College of Medicine, The Catholic University of Korea, Seoul 06591, Korea; alwaysmine8@gmail.com (J.-H.H.); sunnydayzz9292@gmail.com (H.-J.Y.); mrbangga@catholic.ac.kr (C.-H.B.); jylee@catholic.ac.kr (J.-Y.L.); 96015367@cmcnu.or.kr (Y.-M.P.); 2 Department of Business Management, Kwangwoon University, Seoul 01897, Korea; botoks723@gmail.com (J.-W.Y.); ddiyoo@gmail.com (J.-H.C.)

**Keywords:** atopic dermatitis, barrier, dermatology, epidemiology, infection

## Abstract

According to previous studies, the increased risk of cutaneous infectious disorders in patients with atopic dermatitis (AD) is related to impaired epidermal function, abnormal systemic immune function, and lower antimicrobial peptides. In this study, we analyzed the association between AD and cutaneous infectious disorders in the real world using sequential pattern mining (SPM). We analyzed National Health Insurance data from 2010–2013 using SPM to identify comorbid cutaneous infectious diseases and the onset durations of comorbidities. Patients with AD were at greater risk for molluscum contagiosum (adjusted odds ratio (aOR), 5.273), impetigo (aOR, 2.852), chickenpox (aOR, 2.251), otitis media (aOR, 1.748), eczema herpeticum (aOR, 1.292), and viral warts (aOR, 1.105). In SPM analysis, comorbidity of 1.06% shown in molluscum contagiosum was the highest value, and the duration of 77.42 days documented for molluscum contagiosum was the shortest onset duration among all the association rules. This study suggests that AD is associated with an increased risk of cutaneous infectious disorders. In particular, care should be taken regarding its high relevance with impetigo, molluscum contagiosum, and otitis media, which may help in preventing AD from worsening through appropriately preventing and managing the condition.

## 1. Introduction

Atopic dermatitis (AD) is a relatively common, relapsing, chronic inflammatory skin disorder, and its global prevalence is 15–30% in children and 2–10% in adults [1,2,3]. AD has been linked to impaired function of the epidermal barrier and abnormality of the skin innate immune response [4,5]. AD patients may experience chronic itching; sleep disturbances; and impairments in daily, school, and work life; and they are also more susceptible to infections. According to previous research, the increased risk of cutaneous infections in AD relates to impaired epidermal function, abnormal systemic immune function, decreased antimicrobial peptides, and the use of immunosuppressive agents for AD management [6].

Compared to other association rule mining methods, sequential pattern mining (SPM) is a well-designed method for analyzing multiple sequential data with frequent association rules [7,8]. SPM is based on and usually performed in the retail industry to analyze transaction history at the point of sale. In the medical field, SPM could be applied to estimate frequent patterns or behaviors, and it is also useful in identifying signs of concomitant diseases when there are multiple disease claims in a patient’s medical record.

Many reports have demonstrated that AD patients are more susceptible to cutaneous infectious disorders than healthy controls are. However, no studies have evaluated the relationship between the two disease groups using SPM, and there are few studies assessing the time to the onset of these cutaneous infectious disorders after the diagnosis of AD. The aim of our study was to investigate the association between AD and infectious diseases in the real medical environment by SPM using national health data.

## 2. Materials and Methods

### 2.1. Data Source and Study Population

We obtained population-based data from the Korea Health Insurance Research and Assessment Agency (HIRA) from January 2010 to December 2013. The entire Korean population is covered by the National Health Insurance Service (NHIS). In 2013, 97.2% (*n* = 49,989,620) of the population was covered by the NHIS database, and the remaining 2.8% (*n* = 1,458,871) was covered by the healthcare support system. HIRA is under the NHIS, and HIRA claims that the data provide a relatively standardized, stratified sample of 1 million people (*n* = 1,062,018) from the NHIS database, which uses International Classification of Disease, 10th Revision, Clinical Modification (ICD-10-CM) codes. We extracted the study population from the HIRA claims database and anonymized all the data, which were provided to the researcher after blinding the patients’ personal information. This study was approved by the ethics committee of Seoul St. Mary’s Hospital, the Catholic University of Korea (approval no. KC19ZESI0664).

### 2.2. Identification of AD and Cutaneous Infectious Disorders

Patients with AD and cutaneous infectious disorders were identified using ICD-10-CM codes (AD (L20), impetigo (L01), eczema herpeticum (EH; B00.0), chickenpox (B01), herpes zoster (B02), viral warts (B07), molluscum contagiosum (B08), viral conjunctivitis (B30), and otitis media (H65 + H66)). AD patients were defined as those who had been diagnosed with ICD-10 code L20. Patients previously diagnosed with at least one infectious disorder were excluded to evaluate the incidence of cutaneous infectious diseases newly arising in AD.

### 2.3. Statistical Analysis

We compared the occurrence of each cutaneous infectious disorder between patients with AD and a control group using logistic regression analysis. Gender- and age-adjusted odds ratios (aORs) and 95% confidence intervals [9] were obtained using a multivariable logistic regression model. Data were analyzed using SAS Enterprise Miner version 13.2 (SAS Institute, Cary, NC, USA). A value of *p* < 0.05 was considered to be statistically significant.

### 2.4. SPM

We used SPM to evaluate the relationship between AD and cutaneous infectious disorders by time differences, and the parameters for SPM were based on the confidence values, which were defined as the probabilities of occurrence, and the duration values, which were defined as the average occurrence times. In this analysis, age, sex, ICD-10 codes, and dates were used as variables, and we used SAS Enterprise Miner version 13.2 (SAS Institute) to identify the sequential patterns of diseases.

## 3. Results

### 3.1. Baseline Characteristics of the Study Population

From a total population of 1,062,018 subjects sourced from the HIRA database, 70,205 AD patients were included and analyzed (Table 1). The total number of male AD patients was 32,443 (46.21%). The number of patients aged 0–4 years, who were most common, was 18,465, while patients aged 5–9 years were the second-most common and numbered 9814.

### 3.2. Risk of Cutaneous Infectious Disorders in AD Patients

We evaluated the frequencies and risks of cutaneous infectious disorders, namely, impetigo, EH, chickenpox, herpes zoster, viral warts, molluscum contagiosum, viral conjunctivitis, and otitis media among patients with AD (Table 2). According to the multivariate logistic regression analyses of the association between AD and each cutaneous infectious disorder, patients with AD were at greatest risk for molluscum contagiosum (aOR, 5.237; 95% CI, 5.128–5.422). Furthermore, patients with AD showed a higher risk for impetigo (aOR, 2.852; 95% CI, 2.766–2.942), chickenpox (aOR, 2.251; 95% CI, 2.131–2.377), otitis media (aOR, 2.243; 95% CI, 2.194–2.292), EH (aOR, 1.292; 95% CI, 1.156–1.444), viral warts (aOR, 1.1.5; 95% CI, 1.048–1.165), and viral conjunctivitis (aOR, 1.099; 95% CI, 1.077–1.122) compared to controls. The risk of herpes zoster was lower in patients with AD compared to controls (aOR, 0.422; 95% CI, 0.4–0.445).

### 3.3. Sequence Patterns between AD and Cutaneous Infectious Disorders

**Figure 1:**
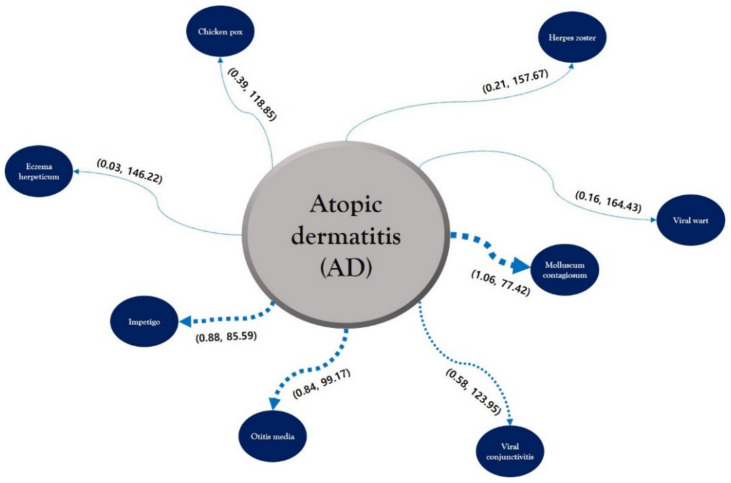
Probability of confidence (%) and time to onset (days) for the sequence patterns between atopic dermatitis and cutaneous infectious disorders. The first number in parentheses indicates the probability of confidence (%), and the second number in parentheses indicates the time to onset (days).

Sequence patterns between AD and cutaneous infectious disorders, and the time to onset were extracted through SPM (Figure 1). The numbers in parentheses in Figure 1 indicate the data for each disease, while the comorbidity association rule “AD ⇒ cutaneous infectious disorders” represents the percentage of patients with the specified cutaneous infectious disorders among all patients with AD. Lastly, the term duration indicates the time (average number of days) that it takes for a patient with AD to be diagnosed with an infectious disorder.

The comorbidity of 1.06% for the association of AD ⇒ molluscum contagiosum was the highest value among all the association rules, followed by 0.88%, 0.84%, 0.58%, 0.39%, 0.21%, 0.16%, and 0.03% for the association rules of impetigo, otitis media, viral conjunctivitis, chickenpox, herpes zoster, viral warts, and EH, respectively. The mean duration of 77.42 days for the association rule of AD ⇒ molluscum contagiosum was the shortest onset duration, followed by 85.59, 99.17, 118.85, 123.95, 146.22, 157.67, and 164.43 days for the association rules of impetigo, otitis media, chickenpox, viral conjunctivitis, EH, herpes zoster, and viral warts, respectively.

## 4. Discussion

This nationwide population-based study evaluated the comorbidity relationship between AD and cutaneous infectious disorders in different age groups, including both children and adults. Although prior studies have evaluated the correlation between AD and cutaneous infectious disorders, most were surveys and included small numbers of subjects, and even previous large-scale studies often included only patients of a certain age group. Notably, a new method called SPM was used here to evaluate the relationship between AD and cutaneous infectious diseases. We reaffirmed the associations with previously well-known diseases, such as molluscum contagiosum, impetigo, viral conjunctivitis, viral warts, and eczema herpeticum. Furthermore, we identified associations between AD and chickenpox and herpes zoster, respectively, which have rarely been studied. Unlike in other studies, the average period of time from AD diagnosis to the first diagnosis of cutaneous infectious disease was analyzed in this investigation. In our study, the percentage of new-onset cutaneous infectious disorders in patients with AD was defined as confidence. As the value of the parameter “confidence” is similar to that of comorbidity in epidemiology, we considered the value of confidence to also serve as a value of comorbidity [7,8]. Moreover, the parameter “onset duration” is, in other words, the time to first diagnosis [8].

According to our results, there was a significantly greater risk of cutaneous infectious disorders in patients with AD than that in individuals without AD. Among the cutaneous infectious disorders considered here, molluscum contagiosum carried the highest risk in AD patients compared to in controls. Furthermore, the duration of time to first diagnosis of cutaneous infectious disorders, especially molluscum contagiosum, was relatively short in patients with AD, namely, approximately 2 months after the diagnosis of AD. Molluscum contagiosum, impetigo, and otitis media showed relatively high confidence values and were also diagnosed within a relatively short period of about 2–3 months. The confidence in viral conjunctivitis, chickenpox, herpes zoster, viral warts, and eczema herpeticum was relatively low, and the average interval for diagnosis took about 3–6 months. Combining these findings with logistic regression analysis, a greater risk of cutaneous infectious disorders was observed in AD patients after age/sex adjustment. However, herpes zoster was observed to have a lower risk in AD patients compared to controls. The correlation between the two diseases and the time to first diagnosis of cutaneous infectious disease may involve the effect of the AD itself or the effect of the drug(s) used for AD. Moreover, it is still not clear whether the average time to first diagnosis of viral conjunctivitis, chickenpox, herpes zoster, viral warts, and eczema herpeticum is actually a late-onset or delayed diagnosis. To clarify the cause affecting the average time to first diagnosis of cutaneous infectious disorders and its mechanism, further research might be needed.

According to previous reports, AD patients may be predisposed to cutaneous infectious disorders. Among them, bacterial infection is suggested to be the most common infection type, and *Staphylococcus aureus* is by far the most common bacterial infection in AD [10,11]. The suggested mechanism for increased bacterial infection rates is barrier dysfunction, immune dysregulation, low antimicrobial peptides, increased bacterial colonization, and the use of immunosuppressant medications for the management of AD [4]. According to our study, AD patients were at 2.8-fold and 1.75-fold higher risks for impetigo and recurrent ear infections, respectively, than the controls were, resembling the results of previous studies. The confidence values of impetigo and otitis media were 0.88 and 0.84, and the onset duration was about 3 months for both. A previous cohort study from the United States that evaluated 3 million subjects < 18 years of age showed that impetigo developed 55% more often in AD patients than it did in the controls after adjusting for age and sex [12]. Moreover, a previous study suggested that AD in children is related to recurrent ear infections as well as other bacterial disorders [4,13].

According to the results, the rates of viral infections, such as molluscum contagiosum, viral warts, herpes simplex virus (HSV) infection (especially EH), chickenpox, and viral conjunctivitis are increased in AD patients [12,14,15]. Epidermal skin barrier defects, innate and adaptive immune dysfunction, decreased antimicrobial peptides, increased skin pH, and Th2 cytokines found in these patients are considered to be enhancing factors for their increased risk of viral infection [16,17,18]. In addition, reports suggest that patients with AD not only have more frequent viral infections, but also more severe and widespread disease than individuals without AD do [14,19]. In our study, the viral infection with the greatest risk in AD patients was molluscum contagiosum, which showed a 5.2-fold increase in risk, with a comorbidity of 1.06% and an onset duration of 77.42 days, which was the shortest onset duration. Several other reports also observed an increased prevalence of molluscum contagiosum in AD patients [15,20,21]. Meanwhile, the risk of viral warts was 1.105 times greater in AD patients than that in the controls, the confidence was 0.16%, and the onset duration was approximately 5–6 months after the AD diagnosis. It is suggested that warts can spread more easily in the setting of AD [19]. The confidence of EH was 0.032%, which was slightly lower than that of other infectious disorders; nevertheless, the risk of EH in AD patients was 1.292 times higher than that in the controls. EH, which is caused by HSV, is a potentially serious complication of AD that presents as generalized vesicles with general symptoms, such as viremia, fever, and lymphadenopathy. HSV exposure is common in the general population, with the virus being present in approximately 60% of the adult population and 20% of children [22]. However, according to other studies, only 3% of AD patients develop EH; compared to AD patients without EH, patients with AD who have EH also seem to have more severe AD, an earlier onset of AD, and increased rates of accompanying atopic diseases [6,17]. Although the comorbid presentation of EH in AD patients is low, as the clinical manifestations of EH tend to appear to be more widespread and severe, we should always keep in mind the seriousness and risk of EH in AD patients.

Our results show that the risk of chickenpox was increased by 2.251-fold in patients with AD compared to patients without AD, and the confidence of chickenpox was 0.39%. Coinciding well with our results, previous studies of pediatric and adult AD patients also reported that the risks of chickenpox were 1.19 times and 1.31 times higher than those in individuals without AD [4,16]. In the case of herpes zoster, our results indicate that AD patients have a 58% lower risk than that of the controls, and the confidence was 0.213%. A previous population-based case–control study involving children demonstrated that the risk of herpes zoster is increased in asthma patients, which is contrary to our data [23]. Although there are few studies on the relationship between AD and varicella-zoster virus infection, the weakened skin barrier function and use of immunosuppressants in AD patients might increase the risk of chickenpox, which is transmitted through direct contact or air. A previous study suggested that AD patients with chickenpox might have longer overall disease duration, more severe disease course, and much higher rate of bacterial superinfection compared to control subjects [24]. In addition, previous research demonstrated that chickenpox vaccination could reduce the complications of chickenpox [25]. However, in Korea, since the chickenpox vaccination is a national mandatory vaccination for all children 12–15 months of age, there was a limitation in confirming the possible influence of vaccination in AD and its severity in this study. Further research on the mechanism might be needed to clarify the association and the exact mechanism.

This study has some limitations. First, the study data were collected from the HIRA claims database without direct review of medical records. In addition, the data used in this study lack information on the severity of diseases or confounding factors such as medication status. In addition, since this study is epidemiologic, analyzing medical claims data, we could not confirm the exact mechanism of the association between AD and cutaneous infectious disorders. Second, our study was a retrospective cohort study. In addition, it is also possible to underestimate the diagnosis if the disease is mild, and if the patient does not visit the medical center. In addition, as patients who had visited the hospital and been diagnosed were included in the study, and those who had not visited the hospital were not included, increasing the chance of selection bias. Third, there is no information on specific infectious species; nevertheless, one of the strengths of this study is that we comprehensively analyzed bacterial and viral infections.

The major strength of our study is that the data we used are maintained by the government or public agencies that provide national health information, and are thus standardized and stable. Additionally, we used a large sample, representative of the entire Korean population, and our study has the advantage of comprehensively analyzing all age groups, while most existing studies were conducted by surveys, interviews, or with a small number of subjects or only children or adults. Lastly, for the first time, the correlation between AD and cutaneous infectious diseases was confirmed using a new method (SPM), and comorbidities, risk, and onset duration were analyzed.

In conclusion, our study demonstrates that AD could increase the risk of cutaneous infectious disorders, and we found that most cutaneous infectious diseases were first diagnosed within several months after the diagnosis of AD. For the first time, we analyzed the time duration of first diagnosis of cutaneous infectious disorders in AD patients. Our findings may be meaningful for early diagnosis and the management of AD and accompanying cutaneous infectious disorders. In particular, care should be taken because of the high relevance of AD with impetigo, molluscum contagiosum, and otitis media, and these results may help in preventing the worsening of disease and with appropriate management in AD patients.

## Figures and Tables

**Table 1:** Demographics of the study population.

	Atopic Dermatitis (*n*)
Total	70,205
Age group, years	
0–4	18,465
5–9	9814
10–14	6786
15–19	4852
20–29	3564
30–39	2703
40–44	3176
45–49	2779
50–54	2935
55–59	2586
60–64	2819
65–69	2556
70–74	1837
75–79	1549
80–84	1559
≥85	2225
Sex	
Male	32,443
Female	37,762

**Table 2:** Three-year risk and confidence of cutaneous infectious disorders in AD patients.

	Confidence (%)	Univariate	Age/Sex Adjusted
		OR (95% CI)	aOR (95% CI)
Impetigo (L01)	0.88	3.352 (3.252–3.455) *	2.852 (2.766–2.942) *
Eczema herpeticum (B00.0)	0.032	1.372 (1.229–1.531) *	1.292 (1.156–1.444) *
Chicken pox (B01)	0.387	3.212 (3.044–3.389) *	2.251 (2.131–2.377) *
Herpes zoster (B02)	0.213	0.449 (0.426–0.474) *	0.422 (0.4–0.445) *
Viral wart (B07)	0.159	1.077 (1.022–1.135) *	1.105 (1.048–1.165) *
Molluscum contagiosum (B08)	1.057	7.118 (6.932–7.310) *	5.273 (5.128–5.422) *
Viral conjunctivitis (B30)	0.576	1.161 (1.137–1.184) *	1.099 (1.077–1.122) *
Otitis media (H65 + H66)	0.836	2.546 (2.492–2.602) *	2.243 (2.194–2.292) *

AD, atopic dermatitis; OR, odds ratio; aOR, adjusted odds ratio; CI, confidence interval. * *p* < 0.001.

## Data Availability

The data that support the findings of this study are available from the Health Insurance Research and Assessment Agency (HIRA). Restrictions apply to the availability of these data, which were used under license for this study. Data are available from [https://www.hira.or.kr/] with the permission of HIRA (accessed on 1 December 2018).
